# Supplementary figures and images for: Spatial clustering and common regulatory elements correlate with coordinated gene expression
Source: PLoS Comput Biol. 2019 Mar 1;15(3):e1006786. doi: 10.1371/journal.pcbi.1006786 (PMC6415868; doi:10.1371/journal.pcbi.1006786)

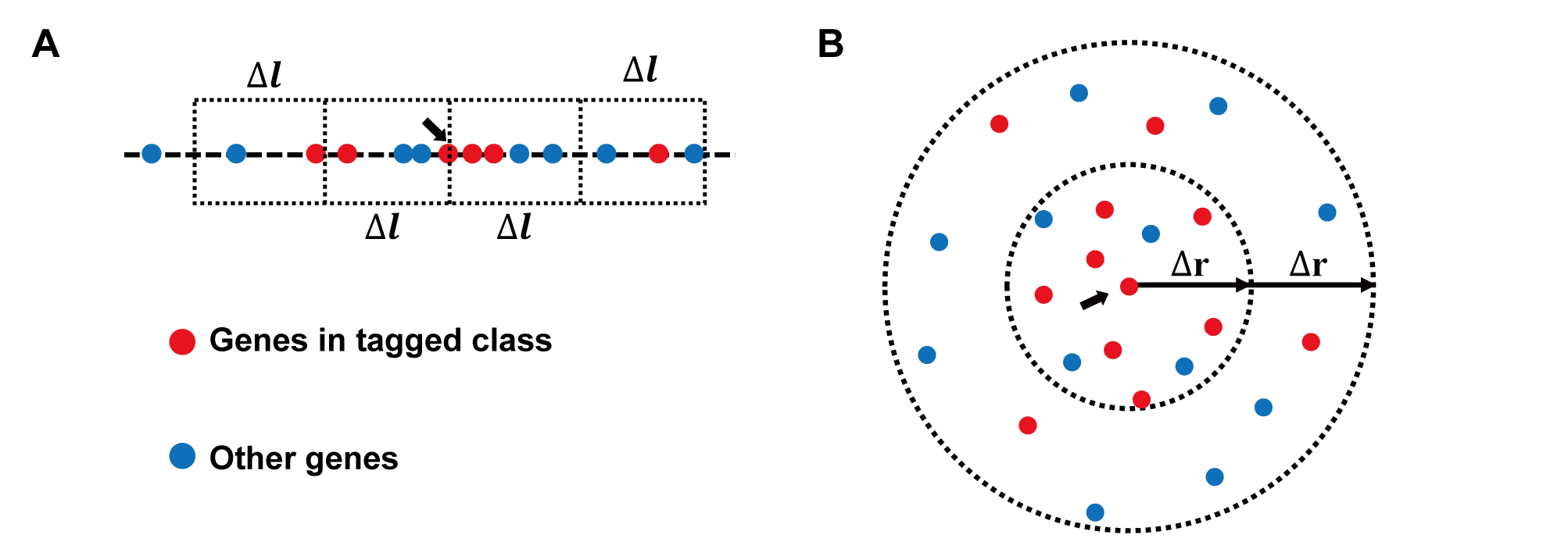

Supplement: S1 Fig — Schematic illustration of the linear (A) and radial (B) distribution functions. (TIF) [file pcbi.1006786.s001.tif]

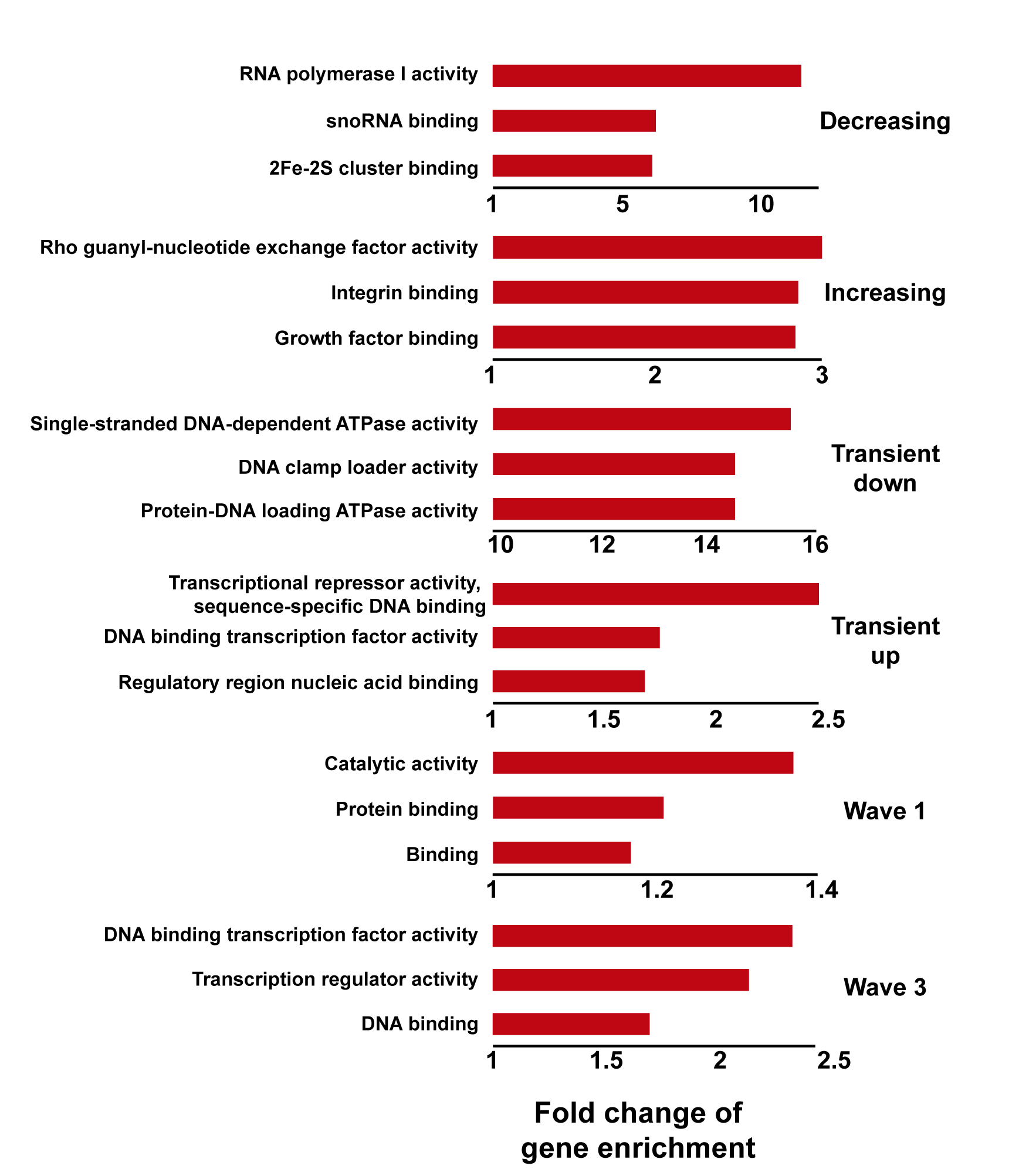

Supplement: S2 Fig — (TIF) [file pcbi.1006786.s002.tif]

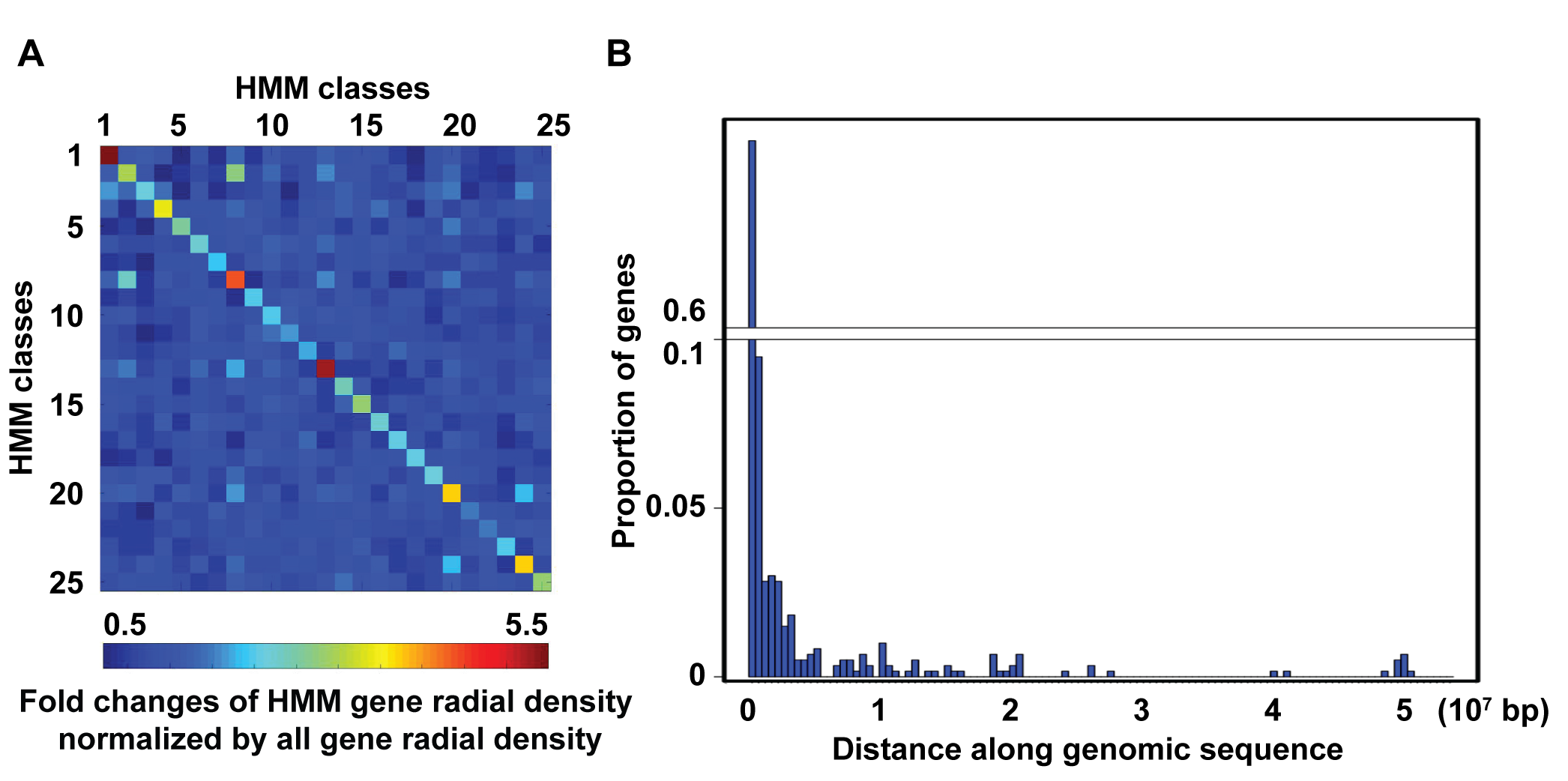

Supplement: S3 Fig — (A) Radial distribution matrix of genes belonging to various HMM classes as normalized by the corresponding average density of all genes around the targeted gene in the first shell (i.e., σαβR(0)/σαAR(0)). (B) Distribution of linear genomic distances between a tagged gene and genes from the same HMM class in the first shell. (TIF) [file pcbi.1006786.s003.tif]

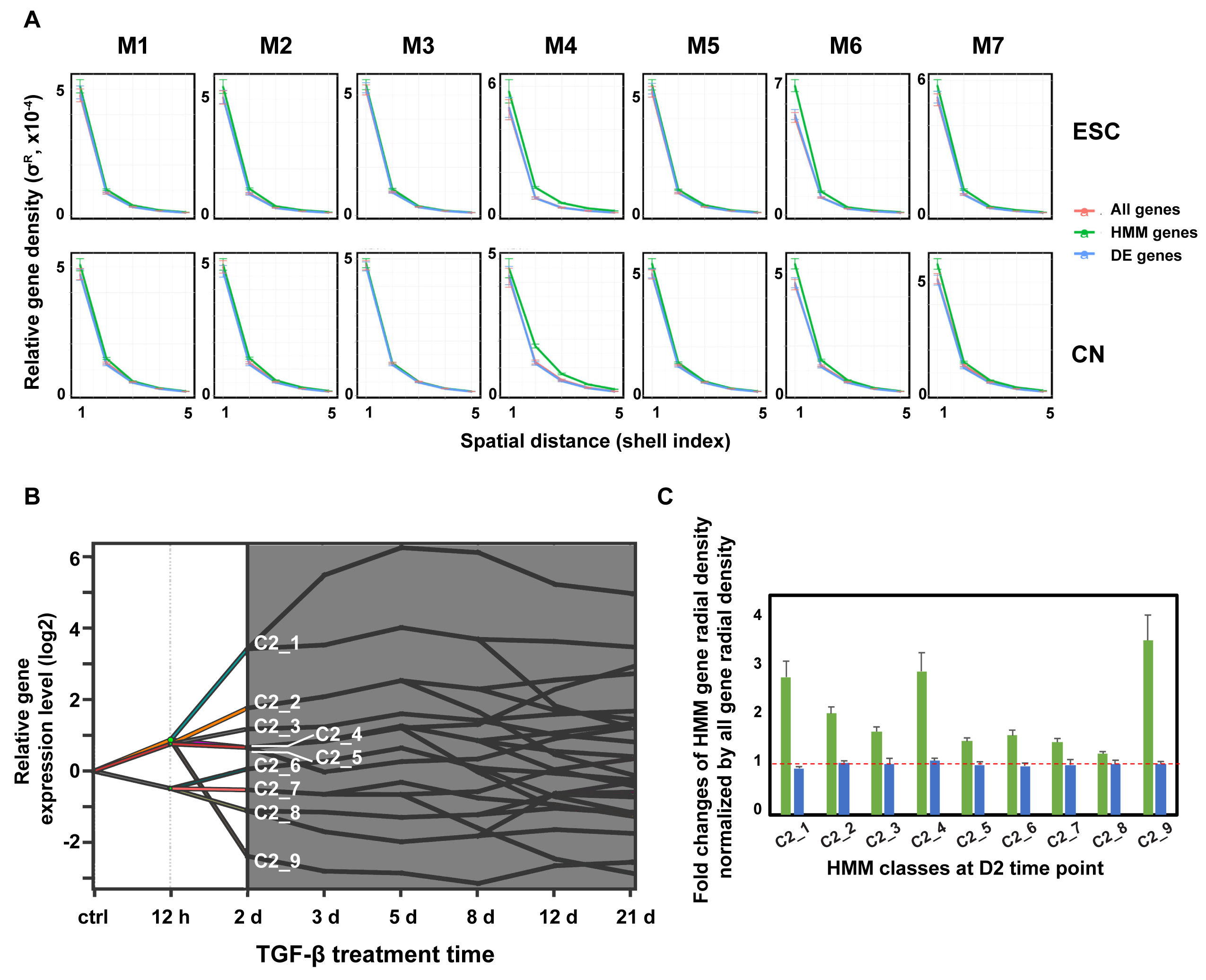

Supplement: S4 Fig — (A) Gene radial distributions, σααR,σαAR and σαDR for the mouse nervous system development, with the shell width Δr approx 60 nm. (B) Eight of the nine HMM classes of TGF-β treated MCF10A identified on day 2 are mixtures of finer HMM classes shown in Fig 3A. (C) Relative gene densities of the HMM classes shown in panel B within the first shell of radial distribution in the first shell, normalized as fold changes with respect to the corresponding average densities of all genes around the targeted genes (the red dashed line), i.e., σααR(0)/σαAR(0) and σαDR(0)/σαAR(0). (TIF) [file pcbi.1006786.s004.tif]
