# Supplementary material for: Spatial clustering and common regulatory elements correlate with coordinated gene expression
Source: PLoS Comput Biol. 2019 Mar 1;15(3):e1006786. doi: 10.1371/journal.pcbi.1006786 (PMC6415868; doi:10.1371/journal.pcbi.1006786)
Supplement: S1 Table — Fig 1 MCF10A cell responses to TGF-β treatment. (A) Schematic diagram of phenotypic transition from epithelial cells to mesenchymal cells in response to TGF-β treatment. (B) MCF10A cells undergo morphological changes in responses to 4 ng/ml TGF-β. (C) PCA clustering of TGF-β treated MCF10A cells reveals distinct gene expression patterns over time. Fig 2 TGF-β induced gene expression changes show distinct temporal patterns. (A) Hierarchical clustering of genes based on temporal gene expression patterns only. (B) Violin plots of distributions of indicated histone modification levels sampled through genes belonging to individual hierarchical clusters. Numbers 1–7 on the x-axis follow the order of gene clusters in panel A. The control group ‘A’ was sampled through all genes. Fig 3 Genes clustered based on both expression patterns and key transcription factors show a correlation between patterns of expression and histone modification. (A) Dynamic regulatory map obtained through the DREM2 analysis. (B) Distribution of indicated histone modification levels sampled through genes belonging to individual HMM classes. Group ‘A’ represents the control group that includes all genes. Fig 4 Genes with similar expression patterns and controlled by the same up-stream regulators show an enhanced tendency to co-localize spatially in the 3D chromosome structure. (A) Heat map shows the numbers of 1-Mb bins containing a given number of genes and TGF-β responding genes. The orange line highlights the bins in which all genes responded to TGF-β treatment. (B) Linear and radial distribution functions of TGF-β-responding genes within two representative HMM classes. We calculated the distribution of genes by sampling three types of gene groups: all available genes (All genes, σαLA and σαAR), genes within an indicated HMM group (HMM genes, σαL and σααR), and genes that showed differential expression during TGF-β treatment (DE genes, σαLD and σαDR). For spatial distance, with the shell width Δr [file pcbi.1006786.s005.docx]

**Table S1 Gene ontology of DREM2 classes.**

| **Class index** | **Enriched functions** | **Fold changes** |
| --- | --- | --- |
| Class 1 | Metalloendopeptidase activity | 16 |
|  | Heparin binding | 15 |
| Class 2 | Calcium ion binding | 4 |
| Class 3 | NA | -- |
| Class 4 | NA | -- |
| Class 5 | NA | -- |
| Class 6 | Protein kinase activity | 3 |
|  | Enzyme binding | 3 |
| Class 7 | NA | -- |
| Class 8 | Proteoglycan binding | 17 |
| Class 9 | Metal ion binding | 2 |
| Class 10 | DNA binding transcription factor activity | 2 |
|  | DNA binding | 2 |
|  | Metal ion binding | 2 |
|  | G-protein coupled receptor activity | 0.13 |
| Class 11 | NA | -- |
| Class 12 | NA | -- |
| Class 13 | NA | -- |
| Class 14 | NA | -- |
| Class 15 | Cargo receptor activity | 13 |
| Class 16 | NA | -- |
| Class 17 | Signaling receptor activity | 0.7 |
| Class 18 | Catalytic activity, acting on a tRNA | 5 |
|  | Structural constituent of ribosome | 5 |
|  | Catalytic activity, acting on DNA | 5 |
|  | RNA binding | 3 |
| Class 19 | Single-stranded DNA-dependent ATPase activity | 40 |
|  | Histone kinase activity | 33 |
|  | ATP-dependent microtubule motor activity, plus-end-directed | 21 |
|  | Cyclin-dependent protein serine/threonine kinase activity | 19 |
| Class 20 | Thiamine pyrophosphate binding | 86 |
|  | RNA binding | 3 |
|  | Protein binding | 1.34 |
| Class 21 | Aminoacyl-tRNA ligase activity | 13 |
|  | Structural constituent of ribosome | 8 |
|  | RNA binding | 4 |
|  | Adenyl ribonucleotide binding | 2 |
| Class 22 | RNA polymerase I activity | 23 |
|  | Proton-transporting ATP synthase activity, rotational mechanism | 23 |
|  | Oxidoreductase activity, acting on the CH-CH group of donors, NAD or NADP as acceptor | 12 |
|  | snoRNA binding | 12 |
| Class 23 | Ligase activity, forming carbon-carbon bonds | 50 |
|  | Coenzyme binding | 5 |
| Class 24 | NA | -- |
| Class 25 | NA | -- |

**NA: no significant enrichment**
